# Ceruloplasmin and Alpha-1-Acid Glycoprotein, but not C-Reactive Protein, Correlate With Serum Ferritin During Various Postpartum/Lactation Periods in Congolese Females

**DOI:** 10.31486/toj.21.0059

**Published:** 2022

**Authors:** Solo R. Kuvibidila, Rajasekharan P. Warrier

**Affiliations:** ^1^ Department of Pediatrics, Division of Hematology/Oncology, Louisiana State University Health Sciences Center, New Orleans, LA; ^2^ Department of Pediatrics, Ochsner Clinic Foundation, New Orleans, LA; ^3^ The University of Queensland Faculty of Medicine, Ochsner Clinical School, New Orleans, LA

**Keywords:** *Acute-phase proteins*, *C-reactive protein*, *ceruloplasmin*, *ferritins*, *inflammation*, *lactation*

## Abstract

**Background:** Serum ferritin usually correlates positively with acute phase proteins (APPs), but limited information is available on this association during various postpartum/lactation periods. The objective of this study was to assess the association between serum ferritin and APPs in Congolese females during different postpartum/lactation periods.

**Methods:** Serum ferritin, C-reactive protein (CRP), alpha-1-acid glycoprotein (AGP), ceruloplasmin (Cp), and transferrin saturation (TS) were measured during various postpartum/lactation periods (0.5 to 6, 6.1 to 12, 12.1 to 18, and 18.1 to 24 months) in 131 Congolese females aged 15 to 45 years**.**

**Results:** Mean serum ferritin concentrations were lower in females in the 0.5- to 6-month postpartum/lactation subgroup than in the other 3 subgroups (*P*<0.05). Mean concentrations of hemoglobin, APPs, and TS were not different among the 4 subgroups. While serum ferritin concentrations correlated with Cp (*r*=0.514) and AGP (*r*=0.795) during the 0.5- to 6-month and the 6.1- to 12-month postpartum/lactation periods, respectively (*P*<0.05), they did not correlate with CRP. Multiple regression analysis suggested that Cp explained 25% of serum ferritin variance in the 0.5- to 6-month postpartum/lactation period (39.3% at 0.5 to 4 months) and AGP explained 60.5% of the variance in the 6.1- to 12-month period (3.7% at 0.5 to 4 months). CRP explained <5% of the serum ferritin variance at these postpartum/lactation periods. APPs explained ≤15.1% of serum ferritin variance at postpartum/lactation periods >12 months.

**Conclusion:** Data suggest that the association between serum ferritin and inflammation is dependent on APP type and lactation time. This association may affect the diagnosis of iron deficiency in lactating females. The positive association between serum ferritin and Cp at 0.5 to 6 months postpartum may be necessary to increase liver iron release and erythropoiesis after childbirth.

## INTRODUCTION

Iron deficiency is a worldwide public health problem that affects females of childbearing age, children, and the elderly.^1^ Assessment involves the measurement of serum ferritin, soluble transferrin receptor, serum iron, total iron binding capacity (TIBC), transferrin saturation (TS), hemoglobin (Hb), red blood cell morphology (hypochromia and microcytosis), and hepcidin (the iron regulatory hormone that controls the release of iron from enterocytes and/or macrophages to the bloodstream).^1-6^ When iron deficiency is severe, Hb levels fall below the cutoff points for age (<11 g/dL for children), sex (<12 g/dL for females and <13 g/dL for males), and certain physiologic conditions such as pregnancy (<11 g/dL for pregnant females).^7^ Of all these measurements, serum ferritin concentration is the most frequently used test in the assessment of iron deficiency. In pure iron deficiency (attributable to inadequate dietary iron, impaired iron absorption, or blood loss), serum ferritin levels are usually reduced below 12 to 15 μg/L with or without a decrease in TS and Hb, while soluble transferrin receptor levels usually increase above 8.3 or 8.5 mg/L.^2,4-9^ In parallel, serum hepcidin levels decrease so that iron release from enterocytes is facilitated.^3,10^ During iron overload, serum ferritin increases above 150 μg/L in females and above 200 μg/L in males.^1^ Although serum ferritin concentrations of 15 to 100 μg/L are usually observed in subjects with adequate body iron stores, these values fall in a gray zone in which iron deficiency cannot be excluded when infection or inflammation is present.^11^ To account for the influence of infection and inflammation, researchers have suggested increasing the cutoff point of serum ferritin to 30 μg/L, 45 μg/L, or 100 μg/L.^4,12-14^ In 2020, the World Health Organization (WHO) set a new cutoff point for serum ferritin of <70 μg/L in individuals with either inflammation or infection.^15^ The uncertainty of the cutoff points of serum ferritin during inflammation or infection may lead to underestimation or overestimation of the prevalence of iron deficiency in individuals who live in areas of the world (including sub-Saharan Africa) where subclinical malaria, intestinal parasites, and other infections (inducers of inflammation) are common.

In 2017, Jorgensen et al reported that serum ferritin did not positively correlate with C-reactive protein (CRP) or alpha-1-acid glycoprotein (AGP) in females at 4 months postpartum.^16^ In fact, mean serum ferritin and serum hepcidin concentrations were lower in females with elevated levels of these acute phase proteins (APPs) than in those with APP levels in the normal range. The observation was unexpected considering that serum ferritin behaves like an APP during inflammation and infection.^11-13^ Jorgensen et al hypothesized that physiologic changes which take place just after childbirth and during lactation may modify the association of serum ferritin and inflammation.^16^

We have published 2 papers on serum ferritin, TS, and soluble transferrin receptors in Congolese lactating and nonlactating females of childbearing age.^17,18^ We analyzed serum ferritin as a function of inflammation using CRP >15 mg/L and AGP >1.2 g/L, as suggested by Engler.^19^ We observed that mean serum ferritin tended to be higher in the females who had inflammation vs those who did not. However, we did not analyze mean serum ferritin concentration or correlation coefficients between APPs and serum ferritin as a function of postpartum/lactation periods.

One of the goals of this study was to determine if poor correlation between serum ferritin and APPs in the postpartum/lactation period was true also for other populations, especially females from sub-Saharan Africa. We therefore tested the hypothesis that elevated blood concentrations of CRP, AGP, and/or ceruloplasmin (Cp) are positively associated with serum ferritin in Congolese lactating females during the 0.5- to 6-month, 6.1- to 12-month, 12.1- to 18-month, and 18.1- to 24-month postpartum/lactation periods.

## METHODS

The original study population included 186 lactating females.^17^ Participants were recruited from the well-baby clinics in rural Bas-Congo in the Democratic Republic of the Congo. The study was approved by the Louisiana State University Health Sciences Center Institutional Review Board in New Orleans, Louisiana, and the Nsundi-Lutete Hospital in Bas-Congo, Democratic Republic of the Congo. The study was conducted according to the guidelines of the Declaration of Helsinki. Participants gave oral consent, and nurses signed the forms before blood drawing.

This study involves a subgroup of 131 females whose postpartum/lactation periods were between 0.5 and 24 months. Other inclusion criteria were availability of data on the concentration of serum ferritin, CRP, and either AGP or Cp, as well as lack of clinical symptoms of any disease at the time of blood drawing. Detailed methods for the measurements of serum ferritin (radioimmunoassay), Hb, serum iron, TIBC (colorimetric methods), and APPs (radial immunodiffusion) have been previously described.^17,20^ TS, expressed as a percentage, was calculated by dividing serum iron by TIBC.

For the definition of inflammation, we used the most recent cutoff points suggested by the WHO panel on assessment of iron status: CRP >5 mg/L and AGP >1 g/L.^4,12-15^ Because the cutoff point for Cp is not clear in the literature—with proposed values of 500 mg/L, 530-540 mg/L, and 600 mg/L—we chose the cutoff point of 500 mg**/**L.^1,21^ We defined the severity of inflammation as none, mild, moderate, and severe with the following number of APPs above normal: APP=0, APP=1, APP=2, and APP=3, respectively. We defined iron deficiency as serum ferritin <15 μg/L in females without inflammation or <70 μg/L in those with inflammation.^15^

### Statistical Analysis

Data were analyzed with Microstat, release 2.0 (Ecosoft Inc).^22^ Because serum ferritin is usually skewed, data were also transformed to decimal logarithm (log) before statistical analysis. Means ± SEM of various indicators of iron status and APPs were compared as a function of inflammation status and postpartum/lactation period by Student *t*-test and/or by one-way analysis of variance (ANOVA). Pearson correlation coefficients between indicators of iron status, specifically serum ferritin and APPs, were calculated to determine which of the markers were strongly associated. Chi-squared test was used to compare the proportion or percentage of females with serum ferritin concentrations suggestive of iron deficiency and who had inflammation vs those who did not have inflammation.

Multiple regression analysis was performed to determine which variable had the strongest influence on serum ferritin concentration. Independent variables included the 3 APPs (CRP, AGP, and Cp), TS, and Hb in the 4 postpartum/lactation periods. The chosen lowest quartile of lactation (6 months) was based on 2 facts: (1) it corresponds to the end of the delayed postpartum period as described by Romano et al^23^ and (2) the WHO and Centers for Disease Control and Prevention recommend that all babies should exclusively be breast-fed during the first 6 months of life and that breastfeeding should continue for 12 to 24 months or longer.^23-25^

To further illustrate the possible temporal change in the association between serum ferritin and APPs, multiple regression analysis was also computed for females in the 0.5- to 4-month, 4.1- to 8-month, and 8.1- to 12-month postpartum/lactation periods. For each statistical test, the level of significance was set at *P*<0.05. A *P* value between 0.05 and 0.1 (0.05≥*P*≤0.1) was considered a trend of difference.

## RESULTS

The mean ± SEM age of the study population was 27.34 ± 0.65 years, and the mean ± SEM of the postpartum/lactation period was 9.95 ± 0.53 months. Table 1 summarizes the data on indicators of iron status and APPs of females by postpartum/lactation period. The mean log serum ferritin concentration of females in the 0.5- to 6-month postpartum/lactation period was significantly lower than the means of females in the other 3 subgroups (*P*<0.05). Mean age and mean concentrations of APPs, Hb, and TS were not significantly different among females in the 4 postpartum/lactation periods.

**Table 1.** Iron Status of Congolese Females as a Function of Postpartum/Lactation Period

	Postpartum/Lactation Period			
Variable	0.5 to 6 months, n=47^†^	6.1 to 12 months, n=36^†^	12.1 to 18 months, n=32^†^	18.1 to 24 months, n=16^†^
Age, years	26.30 ± 1.10	27.44 ± 1.33	28.37 ± 1.22	28.09 ± 2.30
Serum ferritin, μg/L	56.82 ± 6.53	74.01 ± 9.50	74.29 ± 7.99	68.88 ± 8.87
Log ferritin	1.66 ± 0.04^b^	1.80 ± 0.04^a^	1.81 ± 0.04^a^	1.79 ± 0.05^a^
Hemoglobin, g/dL	11.31 ± 0.19	11.37 ± 0.26	11.48 ± 0.21	11.97 ± 0.17
Transferrin saturation, %	21.73 ± 1.89	22.40 ± 2.60	19.82 ± 1.55	18.68 ± 3.36
C-reactive protein, mg/L	4.28 ± 0.07	3.98 ± 0.06	3.96 ± 0.05	5.10 ± 2.43
Alpha-1-acid glycoprotein, g/L	1.28 ± 0.59	1.26 ± 0.11	1.14 ± 0.08	1.17 ± 0.12
Ceruloplasmin, mg/L	437.56 ± 21.06	455.40 ± 16.13	467.81 ± 22.12	454.07 ± 41.24

Note: Data are presented as mean ± SEM.

^†^Maximum sample size.

a>b, *P*<0.05; mean log serum ferritin of females in the ≤6-month postpartum/lactation period was lower than the means of females in the >6-month postpartum/lactation periods.

**Figure 1.**
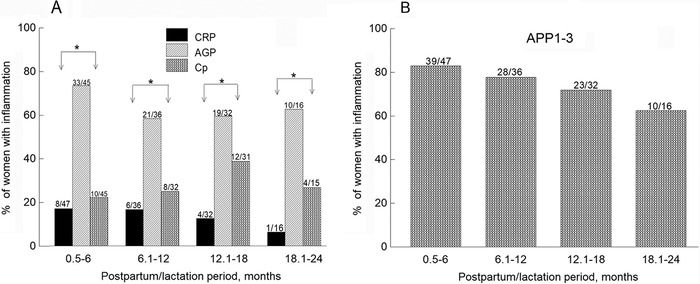
Percentage of females with inflammation assessed by various acute phase proteins (APPs) as a function of postpartum/lactation period. (A) For each postpartum/lactation period, a higher percentage of females had alpha-1-acid glycoprotein (AGP) >1.0 g/L vs those who had C-reactive protein (CRP) >5 mg/L or ceruloplasmin (Cp) >500 mg/L (*P<0.005). (B) Although a higher percentage of females in the 0.5- to 6-month and 6.1- to 12-month postpartum/lactation periods had inflammation compared to those in the 12.1- to 18-month and 18.1- to 24-month postpartum/lactation periods, the differences were not significant by chi-squared test.

For each postpartum/lactation subgroup, a higher percentage of females had inflammation assessed by AGP than by CRP and/or Cp concentrations above normal (*P*<0.005) (Figure 1A). Although differences were not statistically significant, we saw a small trend of a higher percentage of females in the 0.5- to 6-month postpartum/lactation period (73%) vs those in the other 3 postpartum/lactation periods (58% to 62.5%) who had AGP >1 g/L. The overall percentage of inflammation was slightly but not significantly higher for females in the 0.5- to 6-month postpartum/lactation subgroup (83%) vs those in the other 3 subgroups (62.5% to 72%) (Figure 1B).

**Figure 2.**
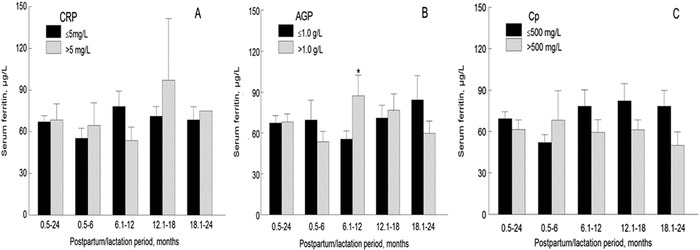
Mean serum ferritin concentrations as a function of concentration of individual acute phase proteins and postpartum/lactation periods. (A) C-reactive protein (CRP). (B) Alpha-1-acid glycoprotein (AGP). (C) Ceruloplasmin (Cp). Mean serum ferritin levels were higher in females with AGP >1.0 g/L than in those with AGP within the normal range in the 6.1- to 12-month postpartum/lactation period (*P<0.05).

In the overall study population and in each of the 4 subgroups, mean serum ferritin concentrations were not significantly higher in females with either CRP or Cp concentrations suggestive of inflammation vs those with CRP and Cp concentrations within the normal range (Figures 2A and 2C). However, mean serum ferritin concentrations in females in the 6.1- to 12-month postpartum/lactation period were significantly higher in those with AGP >1.0 g/L compared to those with AGP ≤1.0 g/L (Figure 2B) (*P*<0.05). No significant difference was observed in mean serum ferritin concentrations in the other 3 subgroups and the overall study population as a function of AGP.

In the overall study population and in females in the 0.5- to 6-month and >12-month postpartum/lactation periods, ANOVA did not detect significant differences among the 3 subgroups defined by inflammatory status.

Mean serum ferritin did not significantly change (increase or decrease) with severity of inflammation (Table 2). In those in the 6.1- to 12-month postpartum/lactation period, mean serum ferritin concentrations were slightly, although not significantly, higher in females with mild inflammation (APP=1 group) than in those without inflammation (*P*<0.1).

**Table 2.** Concentrations of Indicators of Iron Status as a Function of Inflammation (Acute Phase Proteins [APPs]) and Postpartum/Lactation Period in Congolese Females

	Degree of Inflammation		
Postpartum/Lactation Period / Iron Status Indicator	None APP=0	Mild APP=1	Moderate and Severe APP≥2
**0.5 to 24 months**	n=31^†^	n=66^†^	n=34^†^
Serum ferritin, μg/L	70.80 ± 6.82	65.18 ± 6.34	68.18 ± 8.46
Log ferritin	1.788 ± 0.04	1.732 ± 0.03	1.758 ± 0.04
Hemoglobin, g/dL	11.32 ± 2.47	11.43 ± 0.17	11.60 ± 0.18
Transferrin saturation, %	20.17 ± 2.37	21.98 ± 1.64	20.48 ± 2.04
**0.5 to 6 months**	n=8^†^	n=27^†^	n=12^†^
Serum ferritin, μg/L	63.23 ± 18.42	50.55 ± 6.01^b^	66.67 ± 18.50
Log ferritin	1.702 ± 0.105	1.64 ± 0.05	1.701 ± 0.09
Hemoglobin, g/dL	12.01 ± 0.42	10.97 ± 0.25	11.67 ± 0.38
Transferrin saturation, %	22.06 ± 4.90	22.66 ± 2.59	18.94 ± 3.12
**6.1 to 12 months**	n=8^†^	n=22^†^	n=6^†^
Serum ferritin, μg/L	59.75 ± 7.37^*^	81.84 ± 15.09^a^	64.33 ± 9.46
Log ferritin	1.746 ± 0.067	1.833 ± 0.166	1.776 ± 0.083
Hemoglobin, g/dL	10.93 ± 0.71	11.64 ± 0.32	11.04 ± 0.54
Transferrin saturation, %	23.72 ± 6.86	21.19 ± 3.09	24.87 ± 6.96
**12.1 to 18 months**	n=9^†^	n=12^†^	n=11^†^
Serum ferritin, μg/L	78.44 ± 11.04	67.86 ± 13.78^b^	77.91 ± 16.15
Log ferritin	1.843 ± 0.080	1.756 ± 0.074	1.834 ± 0.063
Hemoglobin, g/dL	10.80 ± 0.41	11.78 ± 0.35	11.71 ± 0.28
Transferrin saturation, %	17.00 ± 3.28	22.06 ± 3.24	19.82 ± 1.62
**18.1 to 24 months**	n=6^†^	n=5^†^	n=5^†^
Serum ferritin, μg/L	84.17 ± 17.72	64.40 ± 16.30^b^	55.00 ± 9.10
Log ferritin	1.876 ± 0.093	1.761 ± 0.097	1.709 ± 0.089
Hemoglobin, g/dL	11.82 ± 0.15	12.22 ± 0.51	11.90 ± 0.17
Transferrin saturation, %	17.27 ± 2.46	21.23 ± 4.28	19.16 ± 8.31

Note: Data are presented as mean ± SEM.

^†^Maximum sample size.

^*^P<0.1; mean serum ferritin concentration of females without inflammation (APP=0) in the 6.1- to 12-month postpartum/lactation period was slightly lower than mean serum ferritin concentration of those with mild (APP=1) inflammation.

a>b, *P*<0.05; mean serum ferritin concentration of females with mild inflammation (APP=1) in the 6.1- to 12-month postpartum/lactation period was higher than the concentrations in females in the other 3 subgroups.

In females with mild inflammation (APP=1), mean serum ferritin concentrations were highest in the 6.1- to 12-month postpartum/lactation period and lowest in the 0.5- to 6-month postpartum/lactation period (*P*<0.05). In all of the postpartum/lactation periods, mean concentrations of Hb and TS were not significantly altered (did not decrease) with severity of inflammation.

A higher percentage of females in the 0.5- to 6-month postpartum/lactation period (approximately 66%) vs those in the other postpartum/lactation periods (6.1 to 12 months: 36.11%; 12.1 to 18 months: approximately 47%; 18.1 to 24 months: approximately 44%) had serum ferritin concentration suggestive of iron deficiency (χ^2^=7.93, (df=3), *P*<0.05) (data not shown). In the overall study population, approximately 50% (66/131 females) were iron deficient; 7 of the 66 females were classified as iron deficient because they had Cp concentrations in the range suggestive of inflammation (>500 mg/L).

In the overall study population, serum ferritin positively and significantly correlated with AGP (*P*<0.05) and nonsignificantly with Cp (*P*>0.05), but serum ferritin did not correlate with CRP. Serum ferritin also did not correlate with Hb or TS (Table 3).

**Table 3.** Correlation Matrix Between Indicators of Iron Status and Acute Phase Proteins in Lactating Congolese Females

Postpartum/Lactation Period / Iron Status Indicator	Serum Ferritin	C-Reactive Protein	Alpha-1-Acid Glycoprotein	Ceruloplasmin	Hemoglobin	Transferrin Saturation
**0.5 to 24 months** (n=131^†^)						
Serum ferritin	1	–0.079	0.284*	0.129	–0.021	0.060
C-reactive protein	–0.079	1	0.024	–0.100	–0.025	0.025
Alpha-1-acid glycoprotein	0.284*	0.024	1	0.131	0.069	0.199*
Ceruloplasmin	0.129	–0.100	0.131	1	0.050	–0.085
Hemoglobin	–0.021	–0.025	0.069	0.050	1	0.245*
Transferrin saturation	0.060	0.025	0.199*	–0.085	0.245*	1
**0.5 to 6 months** (n=47^†^)						
Serum ferritin	1	–0.155	–0.137	0.514*	–0.02	–0.131
C-reactive protein	–0.155	1	0.091	–0.181	0.141	0.048
Alpha-1-acid glycoprotein	–0.137	0.091	1	–0.080	–0.325*	0.262
Ceruloplasmin	0.514*	–0.181	–0.080	1	0.083	0.192
Hemoglobin	–0.02	0.141	–0.325*	0.083	1	0.246
Transferrin saturation	–0.131	0.048	0.262	–0.192	0.246	1
**6.1 to 12 months** (n=36^†^)						
Serum ferritin	1	–0.182	0.795*	–0.114	0.009	0.167
C-reactive protein	–0.182	1	–0.138	0.000	–0.220	0.143
Alpha-1-acid glycoprotein	0.795*	–0.138	1	–0.056	0.014	0.218
Ceruloplasmin	–0.114	0.000	–0.056	1	–0.216	–0.062
Hemoglobin	0.009	–0.220	0.014	–0.216	1	0.293*
Transferrin saturation	0.167	0.143	0.218	–0.062	0.293*	1
**12.1 to 18 months** (n=32^†^)						
Serum ferritin	1	0.077	–0.086	–0.191	–0.058	0.226
C-reactive protein	0.077	1	0.230	–0.006	0.106	0.075
Alpha-1-acid glycoprotein	–0.086	0.230	1	0.328*	0.050	–0.051
Ceruloplasmin	–0.191	–0.006	0.328*	1	–0.337*	–0.099
Hemoglobin	–0.058	0.106	0.050	–0.337*	1	0.308
Transferrin saturation	0.226	0.075	–0.051	–0.099	0.308	1
**18.1 to 24 months** (n=16^†^)						
Serum ferritin	1	0.028	–0.243	–0.252	–0.46	–0.221
C-reactive protein	0.028	1	0.057	–0.105	–0.027	–0.489
Alpha-1-acid glycoprotein	–0.243	0.057	1	0.785*	–0.076	0.362
Ceruloplasmin	–0.252	–0.105	0.785*	1	–0.134	0.169
Hemoglobin	–0.46	–0.027	–0.076	–0.134	1	–0.205
Transferrin saturation	–0.221	–0.489	0.362	0.169	–0.205	1

^†^Maximum sample size.

Notes: Data are correlation coefficients (*r*). Values in boldface and followed by asterisks are significantly different from 0 (*P*<0.05). For the 30 females in the 0.5- to 4-month postpartum/lactation subgroup, the correlation coefficients between serum ferritin and acute phase proteins were as follows: *r*=–0.120 for C-reactive protein, *r*=0.597 (*P*<0.05) for ceruloplasmin, and *r*=–0.044 for alpha-1-acid glycoprotein.

Correlation coefficients between serum ferritin and various measurements of inflammation and iron status were also analyzed as a function of postpartum/lactation periods (Table 3). During the 0.5- to 6-month postpartum/lactation period, serum ferritin positively and significantly correlated with Cp (*P*<0.05) but did not significantly correlate with CRP, AGP, Hb, or TS. During the 6.1- to 12-month postpartum/lactation period, serum ferritin positively and significantly correlated with AGP (*P*<0.05) but not with other measurements of inflammation or iron status. During the 12.1- to 18-month and the 18.1- to 24-month periods, serum ferritin did not significantly correlate with any of the APPs.

While AGP positively correlated (*P*<0.05) with Cp in 2 of the postpartum/lactation periods (12.1 to 18 months and 18.1 to 24 months), it did not significantly correlate with CRP in any of the postpartum/lactation periods (Table 3). As expected, Hb positively correlated with TS in the overall study population and in 3 of the 4 postpartum/lactation periods (*P*<0.05) and negatively correlated with AGP and/or Cp (*P*<0.05) at some but not all postpartum/lactation periods.

Multiple regression analysis was performed in 2 steps: (1) including all 131 females and (2) separating females in different postpartum/lactation periods (Table 4). In the overall study population, AGP, Cp, and CRP explained only 6.9%, 0.8%, and 0.6% of serum ferritin concentration variance, respectively. However, when females in the 0.5- to 4-month, 4.1- to 8-month, 0.5- to 6-month, and 8.1- to 12-month postpartum/lactation periods were considered, Cp explained 39.3% (*P*<0.05), 4.94%, 25% (*P*<0.05), and 0% of variance of serum ferritin levels, respectively, compared with 3.7%, 55.84% (*P*<0.05), 1.9%, and 10.1% (*P*<0.05), for AGP.

**Table 4.** Multiple Regression Analysis by Postpartum/Lactation Period With Serum Ferritin as a Dependent Variable

	0.5 to 24 months		0.5 to 6 months		6.1 to 12 months		12.1 to 18 months		18.1 to 24 months	
Independent Variable	*P V*alue	*r* ^2^	*P* Value	*r* ^2^	*P* Value	*r* ^2^	*P* Value	*r* ^2^	*P* Value	*r* ^2^
C-reactive protein	0.423	0.006	0.753	0.003	0.527	0.015	0.712	0.006	0.474	0.107
Alpha-1-acid glycoprotein	0.007*	0.069	0.444	0.019	0.000*	0.605	0.830	0.002	0.591	0.062
Ceruloplasmin	0.369	0.008	0.004*	0.250	0.806	0.002	0.619	0.011	0.390	0.151
Hemoglobin	0.909	0.000	0.523	0.014	0.889	0.001	0.685	0.008	0.132	0.393
Transferrin saturation	0.899	0.000	0.851	0.001	0.904	0.001	0.295	0.050	0.301	0.210
	**0.5 to 4 months**				**4.1 to 8 months**			**8.1 to 12 months**		
	***P* Value**		**r** ^**2**^		***P* Value**		**r** ^**2**^	***P* Value**		**r** ^**2**^
C-reactive protein	0.902		0.000		0.583		0.0153	0.451		0.041
Alpha-1-acid glycoprotein	0.429		0.037		0.0001*		0.5584	0.230		0.101
Ceruloplasmin	0.0004*		0.393		0.320		0.0494	0.992		0.000
Hemoglobin	0.187		0.100		0.362		0.0417	0.694		0.011
Transferrin saturation	0.778		0.005		0.769		0.0044	0.318		0.071

Notes: *r*^2^=variance that predicts serum ferritin concentration. Example: *r*^2^=0.605 corresponds to 60.5% predictive value of alpha-1-acid glycoprotein toward serum ferritin for women in the 6.1- to 12-month postpartum/lactation period. Ceruloplasmin and alpha-1-acid glycoprotein are the 2 acute phase proteins strongly associated with high serum ferritin concentrations. Data for multiple regression analysis for the 0.5- to 4-month, 4.1- to 8-month, and 8.1- to 12-month postpartum/lactation periods were added to further illustrate the weak association between serum ferritin and C-reactive protein. Values in boldface and followed by asterisks are significantly different from 0 (*P*<0.05).

For the other postpartum/lactation periods of interest (6.1 to 12, 12.1 to 18, and 18.1 to 24 months), AGP explained 60.5% (*P*<0.05), 0.2%, and 6.2% of serum ferritin variance compared with 0.2%, 1.1%, and 15.1% for Cp, respectively. For any postpartum/lactation period considered, CRP explained ≤10.7% of serum ferritin concentration variance. Hb and TS explained between 0% and 39.3% of serum ferritin variance.

## DISCUSSION

Serum ferritin behaves as an APP during infection and/or inflammation associated with several chronic diseases, including cardiovascular diseases, rheumatoid arthritis, diabetes, and certain types of cancer.^26-28^ The speculation has been that iron is sequestered in macrophages to deprive it from pathogens and cancer cells or to reduce the production of free radicals that are at the center of many chronic diseases.^26^ However, as observed in this study and reported by Jorgensen et al,^16^ the correlation between serum ferritin and APPs appears to be far from perfect in certain groups of individuals.

The analysis of our data revealed several important observations:

1. As is generally observed in the literature, serum ferritin positively correlated with AGP and slightly with Cp (although not with CRP) in the overall study population of Congolese lactating females.^29-31^
2. The correlation between serum ferritin and individual APPs varies with parturition time; during the first 6 months of lactation, serum ferritin significantly correlated with Cp but not with AGP or CRP. The lack of correlation between serum ferritin and AGP or CRP agrees with the data of Jorgensen et al.^16^ These authors did not measure Cp, so whether there could have been a positive correlation between serum ferritin and Cp is unknown.
3. In the 6.1- to 12-month postpartum/lactation period, serum ferritin positively and significantly correlated with AGP, no longer correlated with Cp, and still did not correlate with CRP. Because Jorgensen et al^16^ studied females only up to 4 months of lactation, confirming or refuting the positive correlation between serum ferritin and AGP in females who have been lactating for more than 6 months is impossible. Correlation coefficients calculated for the 0.5- to 4-month postpartum/lactation period showed that serum ferritin positively and significantly correlated with Cp (*r*=0.597; *P*<0.05) but not with CRP (*r*=–0.120) or AGP (*r*=–0.044). The lack of correlation between serum ferritin and CRP and AGP in the 0.5- to 4-month postpartum/lactation period is also in agreement with the work by Jorgensen et al.^16^
4. Multiple regression analysis that included the 3 APPs confirmed the strong association between serum ferritin and Cp and AGP (although not CRP), but more important, this association was time dependent. As summarized in Table 4, Cp explained 39.3% and 25% of serum ferritin variance during the 0.5- to 4-month and 0.5- to 6-month postpartum/lactation periods, respectively. In contrast, both AGP and CRP explained less than 5% of the variance at both postpartum/lactation periods. However, during the 6.1- to 12-month postpartum/lactation period, AGP explained 60.5% of serum ferritin variance compared with <2% for CRP and Cp. When females in the 4.1- to 8-month postpartum/lactation period were considered, AGP explained 55.84% of serum ferritin concentration variance. Cp, the copper containing and transport protein, is required for iron metabolism; it oxidizes ferrous iron to ferric iron, the form that is transported by transferrin for delivery to various tissues, specifically to the bone marrow.^32^ The authors speculate that the high correlation between serum ferritin and Cp during the first 6 months of the postpartum/lactation period is to ensure iron oxidation and release from storage in the liver for transport to the bone marrow. Iron release would increase erythropoiesis to correct the anemia associated with pregnancy. Later during lactation, Hb levels may already have improved; therefore, the correlation between serum ferritin and Cp becomes poor. Another possible explanation is that later during the postpartum/lactation period, iron metabolism is controlled at the intestinal level by hephaestin, the copper-containing enzyme in enterocytes that oxidizes ferrous iron to ferric iron before its uptake by transferrin and transport to the liver for storage.^32^ If this is the case, then the association between serum ferritin and Cp will definitely diminish. We did not measure hephaestin in our study.
5. The studied females had a relatively low prevalence of acute inflammation, defined by CRP >5 mg/L, with the lowest percentage in the 18.1- to 24-month postpartum/lactation period (6.3%) and the highest percentage in the 0.5- to 6-month (17%) and 6.1- to 12-month (16.7%) postpartum/lactation periods. If AGP is used as a marker of inflammation, between 58.3% and 73.3% of the studied females had chronic inflammation. This finding is not surprising considering that these females live in an area where subclinical malaria and other parasites are common. The lack of correlation between serum ferritin and AGP reported by Jorgensen et al could in part be related to the absence of confounding factors such as malaria and other parasites in their participants.^16^
6. The period of parturition matters when body iron stores are considered: mean serum ferritin concentrations were lower in females in the 0.5- to 6-month postpartum/lactation period vs those in the other 3 postpartum/lactation periods. This finding is easy to understand because body iron stores are reduced or depleted during pregnancy, and repleting them after childbirth takes time. This statement is further supported by the observation that nearly 66% of females in the 0.5- to 6-month postpartum/lactation period (compared with 36% to 47% of those in the 6.1- to 24-month postpartum/lactation periods) had serum ferritin concentrations suggestive of iron deficiency.

Two possible interpretations of the poor correlation between serum ferritin and AGP and CRP in the 0.5- to 6-month postpartum/lactation period are (1) body iron stores are so reduced that moderate inflammation is not sufficient to increase serum ferritin concentrations, and (2) as suggested by Jorgensen et al, pathologic inflammation resulting from infection or other chronic disease is more potent in increasing serum ferritin levels than a physiologic condition such as childbirth.^16,26-31^

The postpartum time-dependent association between serum ferritin and APPs has one important implication on the estimation of the prevalence of iron deficiency in lactating females in communities where inflammation is common. In most studies that involve measurement of markers of iron status, specifically serum ferritin, CRP is the most frequently used marker of inflammation.^26-29,33^ Cp is rarely used but may need to be reconsidered. Females whose ferritin concentrations are in the low range (15-70 μg/L) could be classified as iron-deficient and recommended for iron supplementation. In this study, 7 of 66 iron-deficient females would have been considered iron-sufficient if their Cp concentrations had not been measured.

This study has the following strengths: the measurement of 3 APPs to assess inflammation, the range of postpartum/lactation periods included, and the overall sample size. The principal limitations of this study are the cross-sectional nature of the study; the lack of information about malaria, other parasites, and underlying undiagnosed diseases that could explain the high percentage of females with elevated AGP concentrations; and the small sample size of lactating females in the 18.1- to 24-month postpartum/lactation period.

## CONCLUSION

The new and most important observation of this study is that the association between serum ferritin and APPs during the postpartum/lactation period is time dependent. Cp and AGP explained most of the serum ferritin variance during the 0.5- to 6-month and 6.1- to 12-month postpartum/lactation periods, respectively. During the same postpartum/lactation periods, serum ferritin positively and significantly correlated with Cp and AGP but not with CRP. The APP and time-dependent tendency may affect the diagnosis of iron deficiency in postpartum females with inflammation assessed by one APP. To the best of our knowledge, this study is only the second showing poor correlation between serum ferritin and CRP in females in various postpartum/lactation periods. Two other important observations are that mean serum ferritin levels were lower in females in the 0.5- to 6-month postpartum/lactation period than in those in the 6.1- to 24-month period, and AGP identified more females with inflammation than CRP or Cp. The association between serum ferritin and APPs during various postpartum/lactation periods requires further investigation in communities with a high prevalence of confounding factors, specifically malaria and other parasites.

## Notes

*This article meets the Accreditation Council for Graduate Medical Education and the American Board of Medical Specialties Maintenance of Certification competencies for Patient Care and Medical Knowledge.*
